# Study of the Efficacy and Safety of Contact Lens Used in Trabeculectomy

**DOI:** 10.1155/2019/1839712

**Published:** 2019-07-01

**Authors:** Bin Li, Miaomiao Zhang, Zhen Yang

**Affiliations:** Department of Ophthalmology, The Second People's Hospital of Jinan, 148# Jingyi Road, Jinan 250001, China

## Abstract

**Purpose:**

To investigate the efficacy and safety of soft bandage contact lens used in trabeculectomy.

**Methods:**

This was a prospective, randomized study which enrolled 200 glaucoma patients (200 eyes). Patients were randomized into Group 1, using contact lens after trabeculectomy, and Group 2, without contact lens. The primary outcome measurement was the comparison of success rates at 12 months after surgery. Qualified surgical success was defined as a postoperative intraocular pressure (IOP) value of 6–21 mmHg with or without topical antiglaucoma medication use at the last follow-up visit. Complete success was defined as the IOP between 6 and 21 mmHg without any antiglaucoma medication at the last follow-up visit. Postoperative data included IOP values, best-corrected visual acuity (BCVA), number of antiglaucoma medications, complications related to surgery, and bleb characteristics.

**Results:**

There were statistically significant differences between Groups 1 and 2 in mean IOP values at 3, 6, and 12 months after surgery (*P* < 0.05). The 12-month life table rates for qualified surgical success were 94.7% and 86.3% in Groups 1 and 2, respectively (*P*=0.045). The 12-month life table rates for complete surgical success were 89.6% and 80.0% in Groups 1 and 2, respectively (*P*=0.042). At 12 months after surgery, the mean numbers of antiglaucoma medications were 0.3 ± 0.4 and 0.5 ± 0.6, respectively. (*P*=0.001). At the 12-month visit, the maximal bleb area was significantly different between groups (*P*=0.044), with Group 1 exhibiting a more diffused bleb area. Encysted blebs were observed in 7 (7%) eyes in Group 1 and 17 (17%) eyes in Group 2, with statistically significant differences (*P*=0.030). The 12-month life table rates for qualified surgical success were 94.7% (91 eyes) and 86.3% (82 eyes) in Groups 1 and 2, respectively (*P*=0.045).

**Conclusions:**

Bandage contact lens is a safe and effective device after fornix-based trabeculectomy.

## 1. Introduction

Early postoperative bleb leak (occurring within 1 month after the surgery), which can cause adverse effects such as low intraocular pressure (IOP) and intraocular infection, is a common complication of trabeculectomy [1–5]. Studies suggested that the rates of bleb leaks were between 6% and 59% [5–8]. Bleb leaks are currently treated mainly by surgical sutures, bandages, and wearing bandage contact lenses.

Some studies have reported the use of soft contact lenses for bleb leak [9–14]. Soft bandage contact lens can protect the cornea, accelerate incision healing, and mitigate irrigative symptoms. It also does not affect the eye examinations and drug use. Because of these advantages, contact lens can be traditionally worn after trabeculectomy. Until now, no comparative study has been found about contact lens using in trabeculectomy.

This prospective study was designed to describe the efficacy and safety of soft contact lens in trabeculectomy, with outcomes reported below.

## 2. Materials and Methods

This was a prospective, randomized study and enrolled 200 patients (200 eyes) with primary angle-closure glaucoma (PACG) who underwent trabeculectomy between November 2013 and April 2015. All patients were enrolled after signing an informed consent form. Patients were assigned randomly to Group 1, using contact lens after the surgery, and Group 2, without contact lens. The protocol was approved by the Institutional Ethics Committee of The Second People's Hospital of Jinan and conformed to the tenets of the Declaration of Helsinki.

Patients were included if they were over 18 years old, and had a diagnosis of PACG that could not be controlled with maximal-tolerated medical therapy, or after acute angle closure with at least 12 months follow-up. Patients were excluded if they were followed for less than 12 months, younger than 18 years, with diabetes, and other eye disease. Randomization was determined before surgery according to a block randomization sequence prepared by SAS (version 9.1; SAS Institute Inc., Cary, NC, USA). No patients were excluded after the randomization. Evaluated preoperative data included age, sex, best-corrected visual acuity (BCVA, logMAR Snellen visual acuity), IOP (Goldmann applanation tonometer; AT900; Haag-Streit, Koeniz, Switzerland), and number of antiglaucoma medications.

All surgeries were performed by the same surgeon using the following technique which is the methods of Zhang et al. [15] After surgery, the patients were instructed to stop their preoperative antiglaucoma medications and were prescribed 0.3% tobramycin and 1% prednisolone acetate eye drops 4 times daily for 2 weeks; 1% prednisolone acetate eye drops was decreased to 2-3 times daily for 4 weeks and tapered off over the subsequent 2 to 4 weeks. 1% atropine twice daily was prescribed if IOP was <6 mmHg or if the anterior chamber shallowed.

At the 12-month follow-up, the blebs were graded using the Moorfields Bleb Appearance Grading Scale (MBGS) [16] by a single observer blinded to the respective treatments. An encysted bleb was described as a bleb was walled off by Tenon's capsule and with an IOP >21 mmHg.

Complete success was defined as the IOP between 6 and 21 mmHg without any antiglaucoma medication. Qualified surgical success was defined as a postoperative IOP value of 6–21 mmHg with or without topical antiglaucoma medication. Surgical failure was defined as the IOP value of >21 mmHg, regardless of medication use.

## 3. Statistical Analysis

Data are presented as means ± standard deviations. Independent *t*-tests were used to evaluate between and within groups differences. The Kaplan–Meier method was used to determine survival curves, and differences between them were tested by the log-rank test. A *P* value of 0.05 or less was considered significant. The calculations were performed with the SPSS statistical software (ver. 18.0, SPSS, Inc., Chicago, IL). Statistical significance was defined as a *P* value <0.05.

## 4. Results

The baseline characteristics of the patients are summarized in Table 1. There was no statistically significant difference between the 2 groups in these baseline characteristics. There were 4 patients in Group 1 and 5 patients in Group 2 lost to follow-up.

The IOP differences were significant at 3, 6, and 12 months after surgery (*P* < 0.05; Table 2). At 12 months after surgery, there was significant difference between the 2 groups in the mean numbers of antiglaucoma medications (*P*=0.001; Table 3). At the 12-month visit, there was no statistically significant difference between groups in mean BCVA. (*P*=0.773).

There was no statistically significant difference in MBGS scores for the central bleb area, bleb height, and vascularity between the 2 groups at the 12-month visit (Table 4). But Group 1 exhibited a more diffuse bleb area (*P*=0.044).

Postoperative complications and further surgical interventions are tabulated in Table 5. Two patients with persistent choroidal detachment in Group 2 required surgical intervention (subretinal fluid drainage). Anterior chamber reformation was undertaken in all patients with prolonged postoperative hypotony, which was always accompanied by a prolonged decrease in anterior chamber depth. There was a statistically significant difference between the two groups in the incidence of encysted bleb (*P*=0.030).

**Figure 1:**
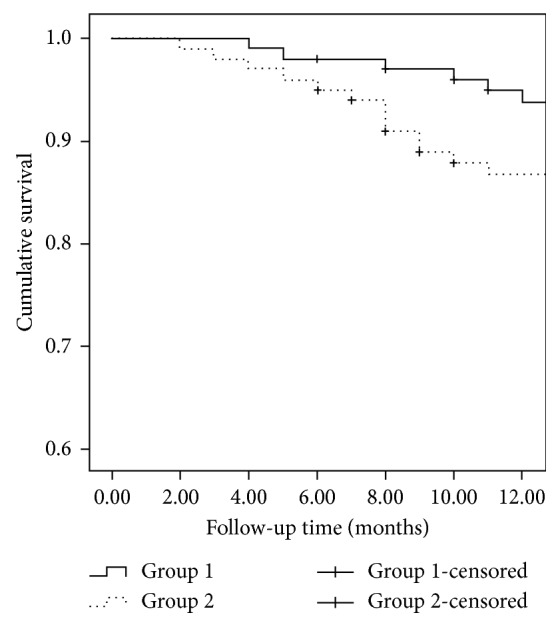
Survival curve with qualifired success defined as IOP between 6 and 21 mm·Hg with or without topical antiglaucoma medication. At 6-month follow-up, the qualified surgical success rates were 97% (97 eyes) and 95% (95 eyes) in Groups 1 and 2, respectively. The 12-month life table rates for qualified surgical success were 94.7% (91 eyes) and 86.3% (82 eyes) in Groups 1 and 2, respectively (*P*=0.045; log-rank test).

**Figure 2:**
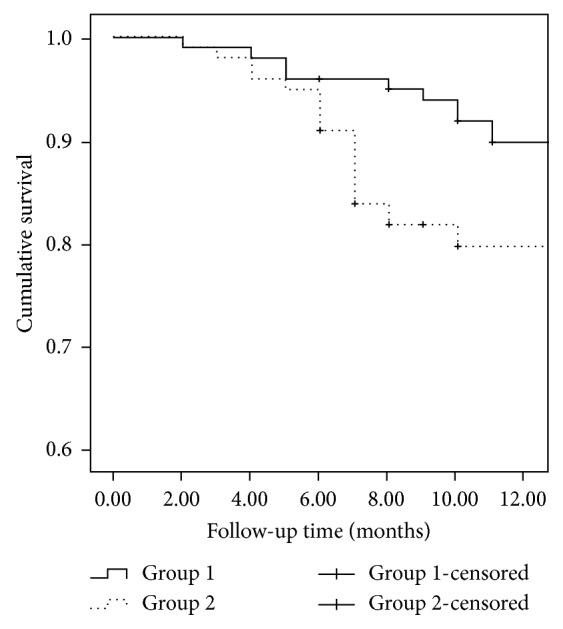
Survival curve with complete success defined as IOP between 6 and 21 mm·Hg without any topical antiglaucoma medication. The complete surgical success rates were 95% (86 eyes) and 91% (91 eyes) in Groups 1 and 2, respectively. The 12-month life table rates for complete surgical success were 89.6% (86 eyes) and 80.0% (76 eyes) in Groups 1 and 2, respectively (*P*=0.042, log-rank test).

At 12-month visit, qualified surgical success was 94.7% (91 eyes) and 86.3% (82 eyes) in Groups 1 and 2, respectively (*P*=0.045; log-rank test; Figure 1). The complete surgical success was 89.6% (86 eyes) and 80.0% (76 eyes) in Groups 1 and 2, respectively (*P*=0.042, log-rank test; Figure 2).

## 5. Discussion

Trabeculectomy is the most common filtration procedure and the gold standard in the surgical treatment of glaucoma. A successful trabeculectomy is generally characterized by formation of a filtering bleb, which is a subconjunctival accumulation of aqueous humor. Bleb leak is a common early complication of trabeculectomy, which may lead to low IOP, choroid detachment, and macular lesions, as well as increased risk of intraocular infection and bleb failure. During trabeculectomy, early bleb leaks are frequently caused by sutures loosening or gap formation after conjunctival edema is alleviated. In addition, antiproliferative agents could increase the risk of a conjunctival bleb leak.

Conventional management of bleb leaks includes surgical sutures and soft therapeutic contact lenses. Some studies have reported the use of contact lenses for bleb leaks [9–14]. Blok et al. reported that the use of a large diameter (20.5 mm) soft therapeutic contact lens promotes the closure of a filtration bleb leak [9]. Fourman and Wiley [11] reported the use of a contact lens (diameter 14.5 mm) to treat a leaking filtration bleb. The bandage contact lenses used in previous studies were of different diameters. So far for the contact lens model, there is no uniform standard. Pure Vision, which was used in this study, is a type of gas-permeable soft contact lens made of silicon-hydrogel and with a diameter of 14 mm. It is readily obtainable at clinics at a low price. The incision of a fornix-based conjunctival flap is made at the corneoscleral limbus so the contact lens could completely cover this type of incision and match the corneal shape, which yields good ocular surface contact and supplies favorable support.

Until now, no comparative study on the use of contact lens in trabeculectomy has been found. Some studies have nonetheless reported the use of soft contact lenses for bleb leaks [9–14]. After more than 12 months, Smith and Doyle [12] and Shoham et al. [13] reported that soft therapeutic contact lenses were a useful tool for early hypotony. Wu et al. reported that the soft bandage contact lens of 14 mm diameter was a safe and efficacious therapy for an early bleb leak following fornix-based trabeculectomy [14]. The results of this study were consistent with the previous findings. In this study, there were more bleb leaks in Group 2. The results can be explained as follows: First, in this clinical trial, the conjunctival incision was completely covered by the contact lens. The contact lens, by slowing down or even stopping the leak flow, allows a restoration of the epithelial barrier function and consequently the closure of the bleb leak. Treatment with the contact lens is an effective alternative in these cases [9]. Second, the contact lens stops the transconjunctival filtration and therefore promotes a reformation of the anterior chamber. At last, the use of the contact lens is a simple method without any side effects, unlike other interventions, such as pressure patching, which is often associated with bleb failure [17].

The 12-month life table rates for qualified surgical success were 94.7% (91 eyes) and 86.3% (82 eyes) in Groups 1 and 2, respectively (*P*=0.045). It can be explained as follows: Early postoperative leakage has an adverse effect on the outcome of trabeculectomy. The continuing production of aqueous and the bathing of the filtration area by the aqueous may assist a possible collagenolytic activity of aqueous and maintain filtration. [18, 19]. In the leakage eye, aqueous flows more easily from the conjunctiva incision wound than it goes under the scleral and conjunctival flap, which can more easily cause some adhesions under the filtering bleb. Fibrosis over the external sclerotomy site after filtration surgery is a far more important cause of failure of the bleb formation and is less easy to prevent [18]. Nonfunctional and flat filtering bleb is more likely to develop in the postoperative bleb leakage eye. This can also explain that there were more encysted blebs in Group 2.

At the 12-month visit, mean IOP values and the number of additional antiglaucoma medications for IOP control were lower in Group 1 than in Group 2 (*P* < 0.05). In the present study, the IOP was measured over contact lens. The use of Goldmann tonometry IOP measurement over a soft contact lens is feasible. A few studies have previously investigated that with a silicon-hydrogel contact lens, the IOP measurement using a Goldmann applanation tonometer was sufficiently accurate [20–24]. Anton et al. reported that even for glaucoma patients, the result of IOP measurements tonometry was sufficiently accurate for clinical practice [24]. Poor agreement has been found between Goldmann applanation tonometer measurements with and without contact lens in glaucoma patients with high IOP [25]. But the results of the report from Gomes should not be used in this study for two reasons: first, inconsistent with the study of Gomes et al., the majority of patients wearing contact lenses in this study were postoperative and with normal IOP; second, these authors concluded that the IOP was higher as measured over the contact lens than without contact lens, which will not change the results of this study [25]. Further studies may provide additional clarification on this finding.

Several studies have reported that bleb leaks occurred more frequently in fornix-based trabeculectomy but cannot be used for limbal-based trabeculectomy [26, 27]. Meanwhile, other reports found no difference about bleb leak between these two groups [28, 29]. A recent systematic review also reported that there was similar efficacy with respect to IOP control and bleb failure (including bleb leak) in both types of conjunctival flap incisions [30]. The contact lenses can be used in fornix-based trabeculectomy, but cannot be used for limbal-based trabeculectomy because the diameter (14 mm) is too short to cover the incision. In this study, we recommend soft contact lenses in all cases of fornix-based trabeculectomy because of high clinical efficacy and low prices.

In this study, daily visual acuity was not significantly influenced by contact lens wear, and no evident ocular discomforts were reported. It is probably because the conjunctival incision and suture were completely covered by the contact lens, thereby easing any eye irritation. The contact lenses were well tolerated by the patients.

This study has some limitations. First, there were individual factors which can affect the wound healing (e.g., immune system), thus influencing the surgical outcomes. Second, even for glaucoma patients, the IOP measurement over contact lens is sufficiently accurate for clinical practice [21]. More prospective studies about the use of contact lens in trabeculectomy are also needed to confirm this conclusion. Third, although the MBGS performed high reproducibility, had higher values for morphologic features, and captured extra vascularity data with probable clinical implications [16] it also has minor deficiencies based on photo grading. At last, although all the surgeries in this study were performed by a surgeon, other things may be important such as the site of application of antimetabolite, the contact with conjunctival border, and the length of the anterior cut side of the scleral flap. We need more relevant research studies about contact lens for trabeculectomy.

The results of this study confirm that the bandage contact lens can effectively reduce the bleb leak after trabeculectomy and improve the success rate of surgery. Contact lens is a safe and effective application after trabeculectomy. However, the clinical outcomes should be further investigated by subsequent investigations with a larger sample size.

## Figures and Tables

**Table 1:** Baseline patient characteristics.

Variable	Group 1 (*n* = 100)	Group 2 (*n* = 100)	*P*
Mean age (years; mean ± SD)	53.27 ± 9.94	54.83 ± 8.39	0.232^*∗*^
Gender (male/female)	66/34	69/31	0.651^#^
Mean BCVA (logMAR, mean ± SD)	0.59 ± 0.21	0.58 ± 0.24	0.754^*∗*^
Number of antiglaucoma medications (mean ± SD)	3.4 ± 0.6	3.5 ± 0.7	0.279^*∗*^
Basal IOP (mmHg; mean ± SD)	29.23 ± 10.37	29.36 ± 10.86	0.931^*∗*^
Type of glaucoma			
Acute PACG	67	65	0.765^#^
Chronic PACG	33	35	

^*∗*^Independent *t*-test. ^#^Pearson *χ*^2^ test. SD: standard deviation, BCVA: best-corrected visual acuity, logMAR: log minimum angle of resolution, IOP: intraocular pressure, PACG: primary angle closure glaucoma.

**Table 2:** Pre- and postoperative mean IOP (mmHg).

Time	Group 1 (mean ± SD)	Group 2 (mean ± SD)	*P*
Basal IOP	29.23 ± 10.37	29.36 ± 10.86	0.931
Postop IOP			
Day 1	8.21 ± 1.62	8.18 ± 1.53	0.893
Week 1	8.95 ± 2.21	8.43 ± 1.91	0.076
Week 2	9.76 ± 2.96	9.25 ± 2.27	0.173
Month 1	10.17 ± 2.98	10.11 ± 2.49	0.877
Month 3	11.14 ± 2.17	11.79 ± 2.13	0.033
Month 6	12.12 ± 2.43	12.79 ± 2.36	0.049
Month 12	12.27 ± 2.69	13.28 ± 2.54	0.006

**Table 3:** Pre- and postoperative antiglaucoma medications (mean ± SD).

Time	Group 1	Group 2	*P*
Basal	3.4 ± 0.6	3.5 ± 0.7	0.279
Month 1^*∗*^	0.1 ± 0.4	0.1 ± 0.5	1.000
Month 3	0.3 ± 0.4	0.4 ± 0.6	0.167
Month 6	0.3 ± 0.4	0.5 ± 0.4	0.001
Month 12	0. 3 ± 0.4	0.5 ± 0.6	0.001

∗: 1 day, 7 days and 15 days after surgery, no glaucoma medication was recorded.

**Table 4:** Bleb morphologic scores at 12 months (mean ± SD).

	Group 1	Group 2	*P* ^*∗*^
Area			
1a	2.1 ± 0.8	2.3 ± 1.1	0.143
1b	3.3 ± 1.1	3.0 ± 1.0	0.044
Height	1.4 ± 0.6	1.5 ± 0.5	0.201
Vascularity			
3a	1.8 ± 0.7	2.0 ± 1.0	0.102
3b	2.7 ± 1.2	2.5 ± 1.1	0.220
3c	1.5 ± 0.6	1.5 ± 0.6	1.000

^*∗*^Mann–Whitney. 1a indicates the central demarcated area of the bleb; 1b, the maximal area; 3a, the vascularity at the central demarcated area of the bleb; 3b, the vascularity at the peripheral part of the bleb; 3c, vascularity at the nonbleb conjunctiva.

**Table 5:** Postoperative complications and postsurgical interventions (%).

	Group 1	Group 2	*P*
Early hypotony (≤1 mo)	6 (6)	16 (16)	0.024
Prolonged postoperative hypotony (≥1 mo)	4 (4)	12 (12)	0.037
Choroidal detachment	4 (4)	5 (5)	0.733
Conjunctival leakage	2 (2)	7 (7)	0.088
Shallow anterior chamber	4 (4)	12 (12)	0.037
Encysted blebs	7 (7)	17 (17)	0.030
Cataract	4 (4)	10 (10)	0.076
Postsurgical interventions			
Conjunctival suture	2 (2)	7 (7)	0.088
Bleb needling	7 (7)	17 (17)	0.030
Anterior chamber reformation	2 (2)	7 (7)	0.088
Subretinal fluid drainage	0	2 (2)	0.155

## Data Availability

The data used to support the findings of this study are included within the article. It is a retrospective study, and some data were unpublished because of patient privacy.
